# High-Performance Ultra-Thin Spectrometer Optical Design Based on Coddington’s Equations

**DOI:** 10.3390/s21020323

**Published:** 2021-01-06

**Authors:** Zhiwei Feng, Guo Xia, Rongsheng Lu, Xiaobo Cai, Hao Cui, Mingyong Hu

**Affiliations:** 1 School of Instrument Science and Opto-Electronics Engineering, Hefei University of Technology, Hefei 230009, China; fengzwchn@mail.hfut.edu.cn (Z.F.); rslu@hfut.edu.cn (R.L.); caixiaobo@mail.hfut.edu.cn (X.C.); cuihao@mail.hfut.edu.cn (H.C.); 2 Academy of Opto-Electric Technology, Hefei University of Technology, Hefei 230009, China; myhu@hfut.edu.cn; 3 Special Display and Imaging Technology Innovation Center of Anhui Province, Hefei 230009, China; 4 National Engineering Laboratory of Special Display Technology, Hefei 230009, China

**Keywords:** optical design, Czerny–Turner spectrometer, cylindrical lens

## Abstract

A unique method to design a high-throughput and high-resolution ultrathin Czerny–Turner (UTCT) spectrometer is proposed. This paper reveals an infrequent design process of spectrometers based on Coddington’s equations, which will lead us to develop a high-performance spectrometer from scratch. The spectrometer is composed of cylindrical elements except a planar grating. In the simulation design, spot radius is sub-pixel size, which means that almost all of the energy is collected by the detector. The spectral resolution is 0.4 nm at central wavelength and 0.75 nm at edge wavelength when the width of slit is chosen to be 25 μm and the groove density is 900 lines/mm.

## 1. Introduction

Spectrometers play an important role in spatially resolved ultrashort pulse measurement, frequency domain optical coherence tomography, identification of substances and remote sensing and other fields [1,2,3,4]. In general, a spectrometer is composed of a slit, a collimating mirror, a focusing mirror, a dispersing component and a detector. With the diversified developing of application, the miniaturization of spectrometers has been put forward. Miniaturization has the advance to increase portability and make it possible to instant measure. Specific applications require miniaturization and miniaturization will lead to more applications. Spectrometers are developing rapidly towards miniaturization with high resolution and high throughput.

Miniaturization of spectrometers has been studied extensively in recent years and many different approaches have been proposed. Freeform surface is a hot topic in optical design because of its more degrees of freedom. A series of methods based on freeform surface to enhance the compactness of spectrometer have been proposed [5,6]. Jacob Reimers et al. introduced freeform surface to eliminate aberrations and designed a freeform surface spectrometer based on the Offner–Chrisp spectrometer [5]. Freeform design was shown to enable 3× spectral-band or 2× spatial broadening or a 5× reduction in volume when compared to a non-freeform counterpart. Feng et al. also presented a compact imaging spectrometer composed of freeform mirrors [7]. In particular, curved prisms were used for dispersing beam. Compared with the conventional system, the volume of the system was reduced by 60% and covered a wide spectrum ranging from 400 nm to 2500 nm. The diffraction grating has been widely used to be the dispersing component due to its high resolution and good stability. The compactness can also be enhanced by utilizing gratings flexibly. Pang demonstrated a compact high-resolution spectrometer by using two plane gratings, a fixed grating and a rotating grating [8]. Light is first diffracted from the fixed grating to the rotating grating, then the rotating grating diffracts the light back to the fixed grating according to the Littrow diffraction method, and finally the light is diffracted a second time by the fixed grating and focused into the fiber. Triple dispersion occurs in the approach and improves resolution and compactness. Li et al. tried to use a plano-concave holographic grating to complete a portable spectrometer design [9]. Planar waveguide is another important method for the miniaturization of spectrometers [10,11]. Faraji-Dana et al. proposed a concept of folded metasurface optics by demonstrating a compact spectrometer. The spectrometer is with a thickness of 1 mm and a volume of 7 cubic millimeters [10]. Using a concave toroidal mirror is also a decent way to improve the miniaturization of spectrometers [12]. Currently, there are microspectrometer products on the market, such as C12880MA and C12666MA developed by Hamamatsu [13]. The overall size is 20.1 × 12.5 × 10.1 mm while the maximum resolution is 15 nm. The methods mentioned above are all aimed at miniaturizing the spectrometer and improving its compactness and portability. These methods are ingenious and remarkable. However, there are still some shortcomings when applied in some situations, such as high resolution but short spectral range, small size but low resolution and good performance but difficult to process.

In many spectrometer configurations, tangential image plane and sagittal image plane vary in positions, resulting in astigmatism [14,15]. The astigmatism is inherent in a spectrometer and cannot be corrected by the spherical mirrors themselves. In most cases, the Czerny–Turner spectrometer has the best structure because its structure and adjustment are simple, and its resolution can be improved and the astigmatism can be compensated by modifying the optical system. The various methods to correct astigmatism in Czerny–Turner spectrometers have been investigated, such as using a toroidal lens [16], using an off-the-shelf cylindrical lens [17] and freeform surface [5,7]. As a precursor to the content of this paper, Wu et al. showed how to eliminate astigmatism [18,19]. Unlike conventional Czerny–Turner spectrometers, the spherical collimating mirror was replaced by two cylindrical mirrors. The equations of positions are constructed by adjusting the distance between two cylindrical mirrors so that astigmatism was eliminated. Compared with the conventional Czerny–Turner spectrometers, astigmatism was well corrected and the root-mean-square (RMS) spot radius is reduced to less than 18 μm.

In this paper, we propose a high-performance ultra-thin Czerny–Turner (UTCT) spectrometer. The spectrometer is designed for improving portability significantly. Building on the previous work done by Wu et al., we trace Coddington’s equations from the ground up and present a complete optical design process, which covers the starting point and the ending point. Coddington’s equations are a direct guide to the design of a high-performance spectrometer.

This paper is structured in 5 Sections. In Section 2, the principle of the method is described, the original Coddington’s equations are given, the complete process of designing is proposed, and the fundamental structure of the spectrometer is Czerny–Turner spectrometer is described. In Section 3, UTCT is designed, the parameters of the optical system are reasonably set and aberrations are corrected in turn (finally, the device F-number is 4 and the spectral range is 670 nm 1130 nm). In Section 4, the design objective is verified in relation to high-throughput, high-resolution and optical size. Section 5 is the conclusion and future expectations about our work.

## 2. Materials and Methods

Spectrometers are usually composed of light source, a slit, a collimating mirror, a dispersion element, a focusing mirror and a detector. Light beam emitted by light source passes through the slit and reaches the collimating mirror. The beam reflected from the collimating mirror is dispersed and then focused on the detector by the focusing mirror. 

**Figure 1:**
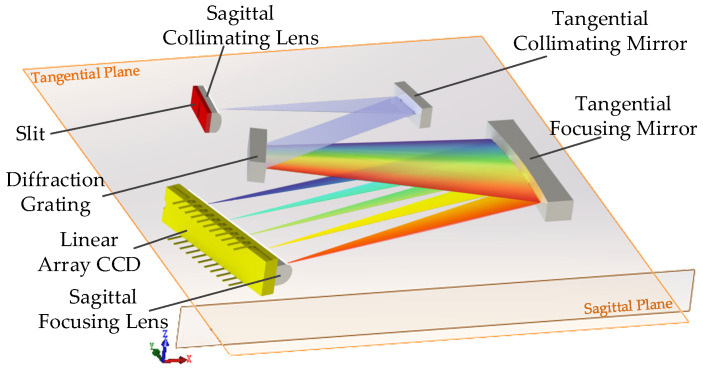
Schematic diagram of an ultra-thin Czerny–Turner (UTCT) spectrometer, consisting of cylindrical lenses and mirrors, where XY plane is the tangential plane of UTCT while XZ plane is the sagittal plane of UTCT.

In a conventional spectrometer, the focal length in sagittal plane of the off-axis spherical mirror is different from that in tangential plane, which is the origin of astigmatism. A practical way to eliminate the difference between tangential and sagittal focus is to split the curvature in the sagittal plane from the curvature in the tangential plane. UTCT is formed with a slit, a sagittal collimating lens, a tangential collimating mirror, a diffraction grating, a tangential focusing mirror, a sagittal focusing lens and a linear array charge coupled device (CCD), as is shown in Figure 1. The slit is located at focal plane of the tangential collimating mirror. The conical divergent light is located at a distance from the tangential collimating mirror, so there will be a certain height in sagittal plane. In order to achieve the ultra-thin design intent, the sagittal collimating lens is placed close to the slit. It means that the height of sagittal plane is getting smaller while the length of tangential plane is no longer than conventional spectrometers. The light beam passes through the sagittal collimating lens and the tangential collimating mirror in turn, so the beam is collimated to be parallel in the tangential plane and the sagittal plane. Then the beam is diffracted by the grating. Finally, the beam is focused on the detector by the tangential focusing mirror and the sagittal focusing lens, which are both cylindrical optical elements.

**Figure 2:**
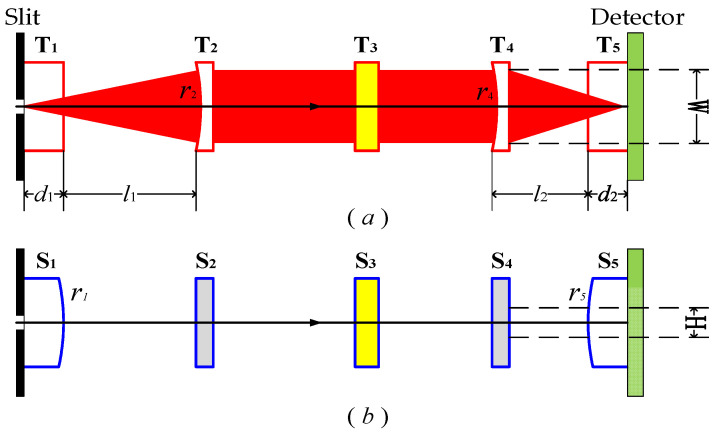
The theoretical schematic diagram: (**a**) UTCT in tangential plane (**b**) UTCT in sagittal plane.

In an optical system a tangential plane and a sagittal plane can be identified. Coddington’s equations are an excellent tool in this case. The relation between object distance (OD) and image distance (ID) can be deduced for tangential plane and sagittal plane using Coddington’s equations. Through this method, the relative position of each optical element can be directly determined, and a high-performance spectrometer can be designed. The theoretical schematic diagrams of UTCT in tangential plane and sagittal plane are shown in Figure 2. T or S represents the optical surface of tangential plane or sagittal plane and the number of the subscript represents the sequence number of the optical plane. T_1_(S_1_) represents the convex surface of the sagittal collimating cylindrical lens. T_2_(S_2_) represents the reflective surface of the tangential collimating mirror. T_3_(S_3_) represents the working surface of the planar diffraction grating. T_4_(S_4_) represents the reflective surface of the tangential focusing mirror. T_5_(S_5_) represents the convex surface of the sagittal focusing cylindrical lens. *r* represents the curvature radius of optical surface and *d*_1_ and *d*_2_ represent the center thickness of the lens. *l*_1_ is the distance between T_1_(S_1_) and T_2_(S_2_) while *l*_2_ is the distance between T_4_(S_4_) and T_5_(S_5_).

According to Coddington’s equations, the relation of OD and ID in tangential plane and sagittal plane can be expressed as

(1) $$\frac{n^{\prime }}{{l^{\prime }}_{s}}=\frac{n}{l_{s}}+\frac{n^{\prime }\cos I^{\prime }-n\cos I}{r}$$

(2) $$\frac{n^{\prime }\cos^{2}I^{\prime }}{{l^{\prime }}_{t}}=\frac{n\cos^{2}I}{l_{t}}+\frac{n^{\prime }\cos I^{\prime }-n\cos I}{r}$$

where *l_s_* and *l_s_′* represents sagittal object distance (SOD) and sagittal image distance (SID), respectively, *l_t_* and *l_t_′* represents tangential object distance (TOD) and tangential image distance (TID), respectively, *I* and *I′* represent the angle of incidence and the angle of refraction, *n* and *n′* represent the refractive index of object space and image space, and *r* represents the radius of curvature of the surface. When Equations (1) and (2) are applied to a mirror, the angle of reflection could be viewed as the angle of refraction. According to Snell’s law, the angle of refraction and the angle of reflection have the same value, but the direction is opposite (*I*′ = −*I*) [20].

The sagittal collimating lens is cylindrical. To reduce the loss of energy, the flat surface of the lens needs to be aligned with the slit and be cling to the slit. The slit is orthogonal to the tangential plane of the cylindrical lens. For convex surface of the sagittal collimating lens, the SID and TID can be expressed as

(3) $$\frac{1}{{l^{\prime }}_{s_{1}}}=\frac{n^{\prime }}{l_{s_{1}}}+\frac{1-n^{\prime }}{r_{1}}=\frac{n^{\prime }}{d_{1}}+\frac{1-n^{\prime }}{r_{1}}$$

(4) $$\frac{1}{{l^{\prime }}_{t_{1}}}=\frac{n^{\prime }}{l_{t_{1}}}$$

where ${l^{\prime }}_{s_{1}}$ and ${l^{\prime }}_{t_{1}}$ are sagittal image distance and tangential image distance, respectively, *d*_1_ is the thickness of sagittal collimating lens. The derivation refers to the principal ray in sagittal plane and the principal ray hits on the cylindrical lens vertically. Therefore, the incidence angle of the convex surface (S1) is 0. It is easy to realize that the lens acts as a plano-convex lens in the sagittal plane and acts as a parallel plate in the tangential plane, as the object distance, $l_{s_{1}}$ and $l_{t_{1}}$ are actually equal, and the numerical value is the thickness of the sagittal collimating lens. The slit is placed at the focus point of sagittal collimating lens and tangential collimating mirror so that the collimated beams can be obtained.

Similarly, the tangential collimating mirror acts as a spherical mirror in the tangential plane and acts as a flat mirror in sagittal plane. It is easy to derive SID and TID in the following equations:

(5) $$\frac{-1}{{l^{\prime }}_{s_{2}}}=\frac{1}{l_{s_{2}}}=\frac{1}{{l^{\prime }}_{s_{1}}}$$

(6) $$\frac{-1}{{l^{\prime }}_{t_{2}}}=\frac{1}{l_{t_{2}}}+\frac{-2}{r_{2}\cos I_{2}}=\frac{1}{{l^{\prime }}_{t_{1}}+l_{1}}+\frac{-2}{r_{2}\cos I_{2}}$$

where $l_{1}$ is the distance between the tangential collimating mirror and sagittal collimating lens, *I*_2_ represents the incident angle of tangential collimating mirror, and *r*_2_ represents the radius of curvature of the tangential collimating mirror. The slit is placed at the focus point so that the collimating beams can be obtained.

The third surface is a planar diffraction grating. The grating can be regarded as a flat mirror in sagittal plane while diffracts the beam in tangential plane:

(7) $$\frac{-1}{{l^{\prime }}_{s_{3}}}=\frac{1}{l_{s_{3}}}=\frac{1}{{l^{\prime }}_{s_{2}}}$$

(8) $$\frac{-1}{{l^{\prime }}_{t_{3}}}=\frac{\cos^{2}i}{\cos^{2}\theta }\frac{1}{l_{t_{3}}}=\frac{\cos^{2}i}{\cos^{2}\theta }\frac{1}{{l^{\prime }}_{t_{2}}}$$

where *i* and *θ* are the incident and diffraction angles of the grating.

The tangential focusing mirror is cylindrical, so the following equations can be obtained:

(9) $$\frac{-1}{{l^{\prime }}_{s_{4}}}=\frac{1}{l_{s_{4}}}=\frac{1}{{l^{\prime }}_{s_{3}}}$$

(10) $$\frac{-1}{{l^{\prime }}_{t_{4}}}=\frac{1}{l_{t_{4}}}+\frac{-2}{r_{4}\cos I_{4}}=\frac{1}{{l^{\prime }}_{t_{3}}}+\frac{-2}{r_{4}\cos I_{4}}$$

where *I*_4_ represents the incident angle, it is also the angle of inclination of the tangential focusing mirror. *r*_4_ represents the radius of curvature of the mirror.

For the last surface, the sagittal focusing lens, the distance can be expressed as

(11) $$\frac{n^{\prime }}{{l^{\prime }}_{s_{5}}}=\frac{1}{l_{s_{5}}}+\frac{n^{\prime }-1}{r_{5}}=\frac{1}{{l^{\prime }}_{s_{4}}}+\frac{n^{\prime }-1}{r_{5}}$$

(12) $$\frac{n^{\prime }}{{l^{\prime }}_{t_{5}}}=\frac{1}{l_{t_{5}}}=\frac{1}{{l^{\prime }}_{t_{4}}-l_{2}}$$

where *l*_2_ is the distance between the tangential focusing mirror and sagittal focusing lens.

The tangential and sagittal image distances of the optical system can be derived as

(13) $$L_{S}={l^{\prime }}_{s_{5}}=\frac{n^{\prime }r_{1}r_{5}d_{1}}{\left(n^{\prime }-1\right)\left(r_{1}+r_{5}\right)d_{1}-r_{1}r_{5}n^{\prime }}$$

(14) $$L_{T}={l^{\prime }}_{t_{5}}=\frac{n^{\prime }r_{2}\cos I_{2}r_{4}\cos I_{4}(n^{\prime }l_{1}+d_{1})}{2r_{2}\cos I_{2}(n^{\prime }l_{1}+d_{1})+\frac{\cos^{2}i}{\cos^{2}\theta }r_{4}\cos I_{4}(2n^{\prime }l_{1}+2d_{1}-r_{2}\cos I_{2})}-n^{\prime }l_{2}$$

Astigmatism can be eliminated if *L_S_* = *L_T_*, so *l*_2_ can be derived as

(15) $$l_{2}=\frac{r_{2}\cos I_{2}r_{4}\cos I_{4}(n^{\prime }l_{1}+d_{1})}{2r_{2}\cos I_{2}(n^{\prime }l_{1}+d_{1})+\frac{\cos^{2}i}{\cos^{2}\theta }r_{4}\cos I_{4}(2n^{\prime }l_{1}+2d_{1}-r_{2}\cos I_{2})}-\frac{r_{1}r_{5}d_{1}}{\left(n^{\prime }-1\right)\left(r_{1}+r_{5}\right)d_{1}-r_{1}r_{5}n^{\prime }}$$

Thus the distance relationship between the optical elements of the spectrometer is derived.

## 3. Results

An UTCT is designed according to the principle in Section 2. The principle is based on Coddington’s equations. In this section, the parameters that were chosen for the proposed spectrometer are further elaborated according to the previously established UTCT optical model. 

The tangential plane of an optical system is more familiar than its sagittal plane. For UTCT, the tangential collimating mirror determines the collection of the incident light and the tangential focusing mirror determines the linear dispersion of the system. In the design case, the F number is chosen to be 4 (the option can completely collect beams with NA of 0.12). *f*_2_ can be inferred to be 40 mm when entrance pupil is 10 mm.

In the design, it is desirable to cover the spectrum all over the photosensitive surface of the detector. The detector we choose is a linear array charge-coupled device (CCD), of which the size is 28.67 mm × 0.2 mm (pixel size is 14 µm × 200 µm and pixel number is 2048). If the spectrum covers the CCD photosensitive surface, *f*_4_ can be derived from the formula

(16) $$f_{4}=\frac{Ld\cos\theta }{\lambda_{2}-\lambda_{1}}$$

where *L* represents the sum of all pixel lengths of the CCD, *d* represents the groove spacing of the grating, *λ*_1_ and *λ*_2_ represent the edge wavelengths at each edge. In the case, the spectral range is 670–1130 nm and the groove spacing is 1.111 μm/line. Diffraction beam of the grating can be determined by the grating equation:

(17) d(sini+sinθ)=mλ

where *m* is the diffraction order and is chosen as 1. In order to restrain the stray light, a certain interval should be kept between the collimating mirror and the focusing mirror in the tangential plane. For this reason, the incident angle *i* is set to 45°, which means the diffraction angle *θ* would be 5.906°. After substituting the values into Equation (16), *f*_4_ is approximated to 68 mm.

Aberration will affect the performance of the spectrometer, and the design method mentioned in Section 2 can effectively avoid the loss of energy caused by astigmatism. If the incident angle of tangential collimating mirror (*I*_2_) is too small, the grating will be very close to the sagittal collimating lens. To avoid this trouble, the *I*_2_ is set to be 14.5°. According to Shafer [21], the coma at the central wavelength can be eliminated by the formula:

(18) $$\frac{\sin I_{4}}{\sin I_{2}}=\frac{r_{4}^{2}}{r_{2}^{2}}\frac{\cos^{3}I_{4}}{\cos^{3}I_{2}}\frac{\cos^{3}i}{\cos^{3}\theta }$$

So *I*_4_ can be obtained, *I*_4_ = 14.968°.

The spherical aberration is inherent in a spherical mirror. The max wavefront aberration (*W_S(max)_*) due to the spherical aberration can be expressed as Equation (19):

(19) $$W_{S(\max)}=\frac{y_{\max}{}^{4}}{8r^{3}}$$

where *y_max_* is the half-aperture of the spherical mirror and *r* represents the spherical radius of curvature of the spherical mirror [22]. Rayleigh principle can be used to determine the effect of the spherical aberration: When the wave aberration produced by the spherical aberration is less than *λ*/4, the effect of the spherical aberration on the imaging can be ignored. So the limitation can be expressed as Equation (20):

(20) $$\frac{y_{\max}{}^{4}}{8r^{3}}\leq \frac{\lambda }{4}$$

When using the method described in this paper, the initial parameters of the system can be derived more accurately. After the radius of curvature is determined, the optimized variables are only the distance between mirrors. The fixed parameters by means of the Coddington’s Equations are shown in Table 1. The optimized parameters are shown in Table 2. The parameter α represents the tilt angle of the detector. The tilt angle of the detector α is set to 0° for the convenience of derivation when the optical system model is built in Section 2. It is changed to be a variable during optimization to compensate for the aberration caused by wide spectral region.

## 4. Discussion

**Figure 3:**
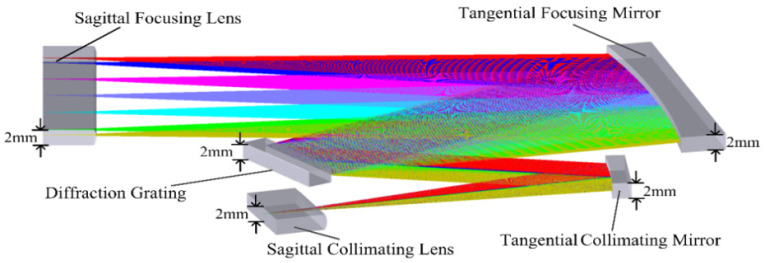
Layout of UTCT, of which the height in sagittal plane is under 2 mm.

In this section we present a design case of UTCT. The initial parameters are calculated in Section 3. The optical system is modeled by using an optical tracing software. Figure 3 shows the layout of UTCT. The height of optical elements in sagittal plane is under 2 mm.

**Figure 4:**
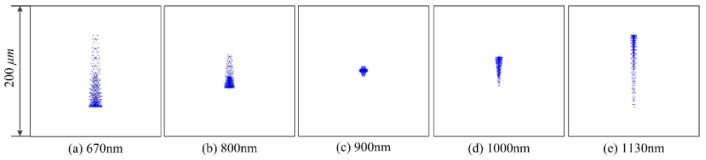
Spot diagrams at several specific wavelengths.

The spot diagram is one of the main evaluation indexes. The performance of UTCT can be evaluated by analyzing the size of spot diagrams. The spot diagrams of several specific wavelengths are shown in Figure 4. It shows that the imaging effect of the center wavelength is excellent as well as the imaging size of the edge wavelength on both sides is kept within 200 μm. As mentioned in Section 3, the height of a pixel is 200 μm. The results show that all of the light beam can be collected by the detector, demonstrating that UTCT is with high throughput.

**Figure 5:**
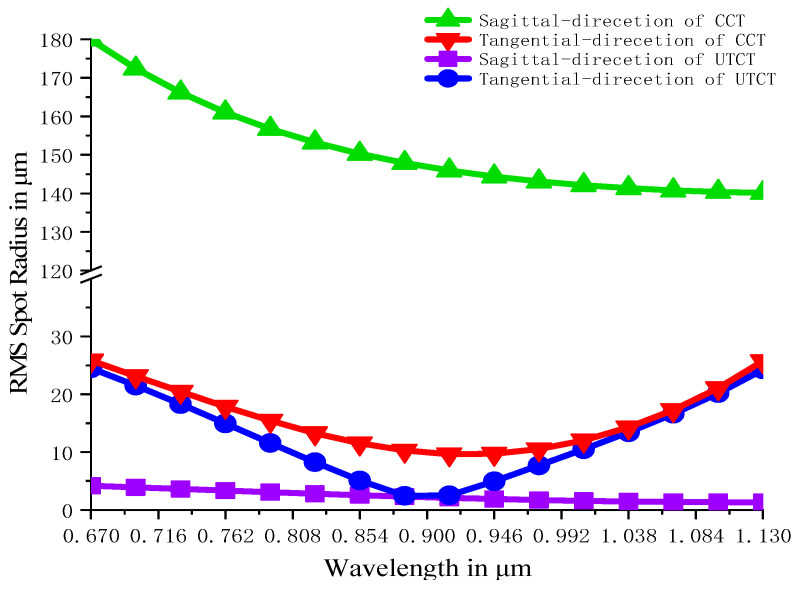
RMS spot radius versus wavelength for UTCT and CCT.

For better comparison, we also design a Czerny–Turner spectrometer with a cylindrical focusing lens (CCT). The sagittal collimating lens is removed and the tangential collimating mirror is replaced by a spherical mirror with the same radius of curvature. Similarly, the tangential collimating mirror is replaced by a spherical mirror with the same radius of curvature and an identical sagittal focusing lens is placed in front of the focal plane. In order to ensure the reasonableness of the comparison, the parameters are optimized in the same way as UTCT. The result of the comparison is shown in Figure 5.

In Figure 5, we associate RMS spot radius with wavelengths to evaluate and analyze the effect of energy concentration. Obviously, the RMS spot radius is reduced to less than 25 μm in the wavelength range 670 nm–1130 nm in tangential plane and the RMS spot radius is less than 5 μm in the sagittal plane as shown. From the contrast of the RMS spot radius between UTCT and CCT in Figure 5, the effect of astigmatism on energy loss is almost eliminated. The results demonstrate that the energy collected by the detector of UTCT will be more concentrated while the resolution remains the same level as CCT. What is more, the thickness of UTCT is obviously get thinner.

**Figure 6:**
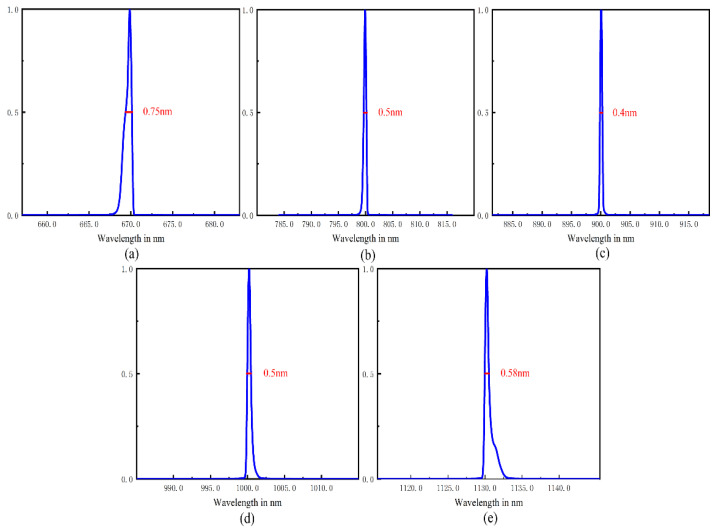
The spectral resolution of the UTCT at typical wavelengths.

Spectral resolution is another important index to evaluate the performance of a spectrometer. Although spectral resolution is defined as the spectral bandwidth detected by a pixel, the effect of slit width should also be considered. In view of this, we calculate the line spread function (LSF) of the ultrathin spectrometer, and convolute the slit and pixel function with LSF. In the whole broadband range, we calculated the LSF of five typical wavelengths and completed the convolution. All results are shown in Figure 6.

The full width at half maximum (FWHM) is used to characterize the spectral resolution of ultrathin spectrometer. In Figure 6, we calculate the FWHM of five typical wavelengths after convolution, and the results show that the resolution of the whole band is within 0.75 nm, and the resolution of the central wavelength is even reach 0.4 nm.

## 5. Conclusions

Based on Coddington’s equations, a high-throughput and high-resolution spectrometer, UTCT, is proposed in this paper. The theoretical analysis of throughput and resolution are detailed in sagittal plane and tangential plane, respectively. The results of the case show that UTCT achieves a full spectral resolution better than 0.8 nm with a minimum thickness of 2 mm. The resolution of the central wavelength (900 nm) is 0.4 nm. Compared with a conventional spectrometer, UTCT not only achieves the level of spectral resolution, but also obtains more concentrated energy at the detector. Although there are more optical elements in UTCT, UTCT is much easier to process than the spectrometer composed by complex surfaces. When compared to a waveguide spectrometer, the size of UTCT may be larger but the resolution may be better and the spectral region may be wider. UTCT reduces the difficulty of processing and promotes the development of compact spectrometer to portable devices. 

## Figures and Tables

**Table 1:** Parameters set by means of the Coddington s Equations (fixed parameters).

Fixed Parameter	Value
*r*_1_ (mm)	2.00
*r*_2_ (mm)	80.00
r_4_ (mm)	136.00
r_5_ (mm)	2.00
*d* (μm/line)	1.111
*d*_1_ (mm)	5.93
*d*_2_ (mm)	5.93
*I*_2_ (°)	14.500
*I*_4_ (°)	14.968
*i* (°)	45.000
*θ* (°)	5.906

**Table 2:** Parameters set by means of the optimization routine.

Parameter	Initial Value	Optimized Value
α(°)	0.000	0.900
*l*_1_ (mm)	34.82	34.80
*l*_2_ (mm)	61.78	61.57

## Data Availability

Data is contained within the article.
